# The Potential Utility of Salivary and Tear Proteomics to Discriminate Sjögren’s Disease from Non-Sjögren’s Sicca

**DOI:** 10.3390/ijms242417497

**Published:** 2023-12-15

**Authors:** Christopher T. George, Biji T. Kurien, R. Hal Scofield

**Affiliations:** 1 McGovern Medical School at UTHealth, Houston, TX 77030, USA; christophergeorgetx@gmail.com; 2 Arthritis & Clinical Immunology Program, Oklahoma Medical Research Foundation, 825 NE 13th Street, Oklahoma City, OK 73104, USA; biji-kurien@omrf.ouhsc.edu; 3 Department of Medicine, University of Oklahoma Health Sciences Center, Oklahoma City, OK 73117, USA; 4 Department of Veterans Affairs Medical Center, Oklahoma City, Oklahoma City, OK 73104, USA

**Keywords:** Sjögren’s disease, proteomics, biomarker, saliva, tears

## Abstract

Sjögren’s Disease (SjD) is an autoimmune disorder associated with decreased saliva and/or tear secretions, resulting in patients reporting dryness in the mouth and eyes. Serum autoantibodies directed against the Ro60/SS-A and La/SS-B autoantigens are a distinctive feature of the disease. Analysis of the saliva and tear proteomes represents one promising alternative method of both classifying and monitoring the condition, and research into salivary and tear proteomics in patients with SjD, with and without sicca, has shown its efficacy and practicality in both clinical and research settings. Studies analyzing the saliva proteomics of SjD patients have generally shown an overexpression of proteins involved in T-cell activation, the immune response, β-2 microglobulin, and the recruitment of pro-inflammatory agents. These studies also show a decrease in or downregulation of proteins involved in salivary secretion. Studies analyzing the tear proteomics of patients with SjD have generally indicated an upregulation of proteins involved with TNF-α signaling, B-cell survival, and the recruitment of pro-inflammatory agents. Studies also note the differential expression of tear protein folding as a hallmark of ocular involvement in this condition. These findings help to elucidate the biochemical relationship between the proteomes of saliva/tear fluids and the general pathophysiology of the gland involved with the pathogenesis of this condition, giving further credence to the potential role of salivary and tear proteomics in the future of diagnosis and treatment for patients with SjD.

## 1. Introduction 

Sjögren’s disease (SjD) is a rheumatic autoimmune disorder that involves the invasion of mononuclear immune cells into exocrine glands, as well as glandular dysfunction, and is responsible for reduced tear and saliva production. As a result, roughly 95% of patients with SjD present with xerostomia and xerophthalmia, or a dry sensation of the mucosal surfaces of the mouth and eyes, respectively [1]. Interestingly, one of the continuing mysteries of this disease is that in many cases the glands look perfectly normal under microscopic examination, even when there is glandular dysfunction. SjD patients also can have extra-glandular manifestations like pulmonary, articular, cutaneous, muscular, renal, and neurological problems [2]. Also, studies estimate a 5–10% lifetime risk of lymphoma [3]. SjD is also associated with fatigue and may be accompanied by a chronic dry cough. The etiology of fatigue in SjD is not currently fully known, but the available evidence suggests that it is a multifaceted disease process. SjD can be symptomatically managed with the use of pilocarpine [4] and cevimeline for mild mucosal drying; however, moderate-to-severe xerophthalmia may require the use of topical cyclosporine [4,5]. In cases of severe xerophthalmia, surgical intervention may be recommended; surgical management primarily consists of performing a lacrimal punctum occlusion, which may aid in the preservation of naturally produced tears on the ocular surface [6]. 

This condition can be further classified into primary Sjögren’s disease (pSjD) and secondary Sjögren’s (sSjD), where pSjD involves no associated diseases, while sSjD is accompanied by other autoimmune diseases such as systemic lupus erythematosus (SLE), rheumatoid arthritis (RA), or systemic sclerosis [7]. The association between SLE and SjD is of particular note, as meta-analyses of the literature have revealed that 14–17.8% of SLE patients also have SjD, and previous studies have shown that sSjD is responsible for the sicca symptoms seen in SLE [8]. 

SjD is characterized by antibodies targeting Ro ribonucleoprotein (RNP) particles containing a 60,000-molecular-weight protein (60 kDa Ro or SS-A). Ro60/SS-A (Sjögren’s syndrome A antigen) associates non-covalently with a minimum of one of four short uridine-rich hyRNAs (human cytoplasmic RNAs). The hyRNAs are associated with the 48,000-molecular-weight phosphoprotein La (SS-B) autoantigen, at least briefly [9]. Anti-Ro and anti-La antibodies can be detected in over 70% and 35% of patients with SS, respectively [10,11].

Proteomic analysis of specific unique biomarkers taken from saliva and tear samples may solve some of the underlying issues that have been observed with the use of serology for traditional SjD diagnosis or research classification. These samples can be readily collected in a noninvasive manner, and the biochemical contents of the samples may reflect pathophysiological changes to the glandular tissue; therefore, proteomics of these fluids may serve as a means to both aid in the diagnosis of the condition and monitor the progression of disease. Thus, previous research in the field of proteomics has been principally focused on identifying the unique protein biomarkers related to pSjD to explore diagnostic capabilities, and to understand whether proteomics may be used in the monitoring of SjD. One important challenge with the use of saliva and tears as biochemical markers is the fact that the proteome of the samples is sensitive to spontaneous degradation, necessitating special methodological approaches for storage. Previous studies have indicated that storing saliva immediately at a temperature of −80 °C, with the addition of protease inhibitors and the removal of mucins through filtration or centrifugation, will allow for minimal proteolysis [12]. Studies regarding tear collection and storage have found that 40 µL of sterile 0.9% saline dispersed onto the superior bulbar and then the inferior fornix of the eye allows for the collection of a tear wash, which can then be stored at −80 °C to prevent proteolysis of the tear proteome [7]. Unfortunately, immediate storage at −80 °C is impractical or impossible in many clinical settings; thus, the utility of saliva or tear proteomics remains in question from a logistical point of view. 

Many patients present with sicca but ultimately are not found to have SjD (non-Sjögren’s sicca, or non-SS). Discrimination of SjD from non-SS is a difficult clinical problem. Therefore, this review article will highlight the recent advances made in the field of proteomics in relation to SjD, especially its use in discriminating SjD from non-SS. 

## 2. Classification Criteria for SjD

Classification criteria are a set of parameters used to assess whether a patient is eligible for participation in a clinical research study; specifically, with regard to clinical studies of SjD. That is, these parameters act as a standardization method to homogenize sample sets across scientific studies produced by different independent research institutions. In 2002, the American–European Consensus Group (AECG) consolidated the literature regarding SjD to create a formal set of criteria regarding the classification of patients for participation in research, and they updated the exclusion criteria to be more stringent in an effort to improve future research’s accuracy [13]. Table 1 summarizes the criteria outlined by the AECG, as well as the rules for classification. 

In 2016, the American College of Rheumatology (ACR), in a combined effort with the European League Against Rheumatism (EULAR), formulated a set of criteria based on specific structured methodology (Delphi). The new criteria merged features of the AECG criteria with prior ACR guidelines in order to create a set of criteria designed for entry classification into clinical trials [14]. Table 2 summarizes the criteria outlined by the ACR/EULAR guidelines for the classification of SjD. 

As detailed in the ACR/EULAR criteria paper [15], the clinical diagnosis of SjD remains a matter of expert opinion.

Much of the recent advancement in SjD’s definition, both in clinical diagnosis and in classification criteria for research, would not have been feasible without the contribution of the Sjögren’s International Collaborative Clinical Alliance (SICCA). SICCA is an international longitudinal collaboration between independent research institutions, ultimately leading to the creation of a biorepository of samples collected from patients with pSjD, sSjD, and non-SS [16]. The result of this effort has allowed for the creation of a biorepository and data registry of 3514 well-defined patients, which was informative in the creation of the aforementioned ACR/EULAR criteria, and for the development of several studies that reviewed the biospecimens collected and catalogued by SICCA for proteomic analysis. In addition to the biospecimens collected, genotyping was performed using the Omni2.5M platform. More recently, the Sjögren’s Genetics Network (SGENE) was established as an international collaboration to better understand the genetic basis for the pathology and variance associated with SjD. Several genome-wide association studies have been performed using genetic data available through these international efforts, primarily through analysis focused on examining single-nucleotide polymorphism associations; however, a comprehensive examination of these studies is outside of the scope of this review [17,18].

## 3. Salivary Protein Profiling

The normal biological function of saliva is ensuring that the mouth remains moist, acting as an antimicrobial, which prevents dental and periodontal disease from occurring. Saliva represents an attractive target for proteomic analysis, as the fluid consists of a wide array of proteins that have been linked in many studies to different pathophysiological changes and can thus be readily used for diagnostic means and to monitor disease progression [19]. This, in conjunction with the readily available means of collection and analysis, some of which have been around since the early 1900s, represents the principal factors behind the large swaths of scientific research aiming to understand the proteomic composition of the fluid. 

Salivary protein profiling involves the identification of proteins that are found in whole and/or parotid saliva samples. This process can be used to identify proteins that are overexpressed, under expressed, absent, or only present in a diseased state, allowing for the identification of biomarkers (Table 3). 

### 3.1. Methodological Approaches Used for Saliva Proteomics

Studies performed regarding the analysis of the proteome of saliva have generally utilized similar methodological approaches. Specifically, bottom–up proteomics with tandem mass spectrometry (MS/MS), a technique with proven efficacy, was used to identify 1939 unique proteins in a study performed on whole and parotid saliva samples [20]. This study was one of the most comprehensive studies regarding the total protein contents of salivary samples, giving further credence to the high throughput benefit of this approach. 

#### 3.1.1. Gel Electrophoresis

A widely used technique for separating proteins is gel electrophoresis. This technique typically involves two-dimensional sodium dodecyl sulfate (SDS)–polyacrylamide gel electrophoresis (2D SDS-PAGE), which separates the various protein isoforms present in a sample. The protein spots generated after staining may be removed/excised from the gel and then characterized by mass spectrometry. Of note with this technique, prevalent salivary proteins such as amylase may require selective depletion before two-dimensional electrophoresis (2DE) in order to enhance the detection of less-abundant proteins [21]. Additional studies have noted the use of high-throughput proteoarrays as an approach for biomarker identification in conjunction with these methods as well [22]. The results of two-dimensional gel electrophoresis can then be tested for reliability with the use of Western blot analysis, in order to verify the presence of the separated proteins within the mixture generated through electrophoresis [23]. 

#### 3.1.2. Mass Spectrometry 

The use of liquid chromatographic methods in conjunction with MS is another commonly used technique in the field of salivary proteomics. This technique is typically prefaced by the fractionation of a whole saliva sample, where the small protein fraction (salivary peptides) are separated by liquid chromatography (LC), while larger protein fractions are digested by trypsin into smaller samples before LC. One study followed this step by the use of online electrospraying with MS/MS using a quadrupole time-of-flight mass spectrometer, where the results were subsequently processed against a human database of protein sequences for analysis [24]. This same study showed the efficacy of such a technique, in that the effort successfully cataloged 309 proteins from whole saliva. 

Two forms of laser desorption/ionization techniques may also be used with mass spectrometry, including matrix-assisted laser desorption/ionization (MALDI) and surface-enhanced laser desorption/ionization (SELDI). MALDI is a technique that involves mixing a sample with a matrix material and applying it to a metal plate for irradiation by a laser. SELDI is a derivative of this technique, using a solid-phase chromatographic separation step, which allows proteins that have attached to the surface to be analyzed via mass spectroscopy. One study showed the usefulness of this technique when used with time-of-flight (TOF) mass spectroscopy, essentially combining the precision of mass spectroscopy and the high-throughput nature of protein arrays [24]. The study concluded that both the monitoring of disease and the discovery of novel biomarkers might be possible with SELDI-TOF. 

### 3.2. Novel Salivary Biomarkers in Sjögren’s Disease

Saliva is secretory product of the salivary glands—one of the histopathological targets of the autoimmune response that is integral to the etiology of SjD. Due to this fact, the proteome of saliva may reflect changes in the pathophysiology of the salivary gland concurrent with the incursion and progression of the condition. Therefore, much of the existing research regarding proteomic analysis of SjD has aimed to identify novel biomarkers associated with the condition, in hopes of providing accurate diagnostic and prognostic criteria for the condition without the reliance on invasive methods. In order to detect novel biomarkers present in saliva, investigators need a database of the normally occurring proteins found in salivary samples. This led the National Institute of Dental and Craniofacial Research (NIDCR) to fund research that was ultimately spearheaded by the University of California at Los Angeles (UCLA), in which a data repository for salivary proteomics was developed: the Salivaomics Knowledge Base (SKB). The SKB allows researchers to compare proteins identified through proteomic analysis of saliva samples to an existing database with all of the known normal proteins readily found in saliva, thus allowing for the identification of novel proteins and peptides [25]. 

#### 3.2.1. Identification of Salivary Biomarkers Using Database Studies

Previous research has focused on distinguishing SjD from non-SS patients, or SjD from healthy controls. The latter is a clinically trivial problem but may lay the groundwork for studies distinguishing SjD from non-SS. One such study utilized DAVID (Database for Annotation, Visualization, and Integrated Discovery) analysis and found that peptidyl-prolyl cis-trans isomerase FKBP1A, β-2 microglobulin, and CD44 antigen were the three most upregulated proteins found within stimulated whole-saliva samples in their studies of pSjD patients relative to non-SS [26]. These proteins are intimately involved in T-cell activation and the inactivation or downregulation of T-cell-suppressive pathways. Additionally, β-2 microglobulin is of particular note, as it is a common biomarker found across many proteomic studies, and previous studies have concluded that, in pSjD, higher levels of β-2 microglobulin and free immunoglobin light chains are linked with increased systemic disease activity [27]. The same study found that β-2 microglobulin was also one of the three most upregulated proteins when analyzing the salivary proteomes of SjD patients and controls, showing that it may be a biomarker that distinguishes pSjD patients from both healthy controls and non-SS patients. Additionally, the extracellular vesicles (EVs) of whole saliva in pSjD patients showed statistically significantly upregulated levels of MVP and NGAL (LCN2) compared to non-SS patients. This study provides an interesting insight into observable trends in the identified proteins discovered; however, this study was conducted with a sample size of 25 experimental subjects and 10 control subjects; thus, the results of the study cannot be used to make generalizable conclusions. Other studies have shown that NGAL has been identified as an upregulated protein in SLE, which may provide discrimination of SLE patients and controls in proteomic analysis [28]. 

#### 3.2.2. Identification of Salivary Biomarkers Using Western Blotting

One study utilizing protein blotting found that pSjD is characterized by a sharp reduction in various secretory proteins, including α-amylases, cystatins, prolactin-induced protein, glyceraldehyde-3-phosphate dehydrogenase, SPLUNC-2, and carbonic anhydrase VI (compared to secondary Sjögren’s syndrome or non-SS) [23,29]. The same study noted an increase in the expression of proteins involved in the autoimmune response of the invading lymphocytes (β-2 microglobulin, rheumatoid factor, and immunoglobulin kappa constant protein), as well as an increase in pro-inflammatory proteins (α-enolase, S100-A7, S100-A9, and lipocalin), compared to secondary Sjögren’s disease and other sicca syndromes. These findings further elucidate the relationship between the proteome of the saliva and the pathophysiology of the gland, as increased levels of autoimmune-related proteins and pro-inflammatory agents are congruent with the pathogenesis of lymphocytes invading the glands and the chronic inflammation associated with the condition, respectively. In addition, reduced levels of secretory proteins can be correlated with reduced saliva secretion by those experiencing xerostomia. An important conclusion from this study that is related to the methodological approach of using proteomics for distinguishing pSjD, secondary Sjögren’s, non-SS, and healthy controls is that a panel of protein markers may serve as a better indicator of the condition rather than focusing on the identification of individual unique biomarkers. While this conclusion is supported by the evidence generated in the study, additional studies must be performed before the reliability of protein panels for distinguishing SjD from those without SjD can be said to be more effective than that of single-protein biomarkers. 

#### 3.2.3. Identification of Salivary Biomarkers Using Mass Spectrometry

Baldini et al. carried out salivary proteomic analysis combining two-dimensional electrophoresis (2DE) and matrix-assisted laser desorption/ionization time-of-flight mass spectrometry (MALDI-TOF-MS) to improve the discriminatory power of a candidate salivary biomarker panel found in pSjD compared to healthy volunteers and non-SS controls. Of the fifteen differentially expressed proteins in pSjD with respect to non-SS, sSJD, and healthy volunteers, the proteins α-amylase precursor, carbonic anhydrase VI, β-2 microglobulin, glyceraldehyde-3-phosphate dehydrogenase (G3PDH), epidermal fatty-acid-binding protein (E-FABP), and immunoglobulin k light chain (IGK-light chain) showed the most significance for discriminating those with SjD from non-SS and healthy volunteers. However, the study’s authors found that pSjD and sSjD shared a greatly similar salivary protein profile [23]. 

One study utilizing LC-MS/MS conducted on whole-saliva samples collected from SjD and non-SS patients found that proteins involved in immunoinflammatory mechanisms were upregulated and secretory protein products were significantly downregulated [30]. Specifically, this study identified neutrophil elastase, calreticulin, and TRIM29 as a protein panel that accurately distinguished pSjD patients from non-SS patients. Another study utilizing a similar methodological approach to identify potential novel biomarkers in saliva from pSjD patients and healthy controls found that lipocalin-2, clathrin assembly lymphoid myeloid leukemia (CALM) protein, progranulin, and calmodulin-like 5 (CALML5) were upregulated [31]. Lipocalin-2 is involved in innate immunity, CALM is a cell signaling protein, and progranulin and CALML5 are both involved in protein processes that activate wound repair [31]. 

The same study analyzed salivary EVs and found that signal-regulatory protein alpha (SIRPA), lymphocyte-specific protein 1 (LSP1), and adipocyte plasma-membrane-associated protein (APMAP) showed statistically significant upregulation. SIRPA and LSP1 are involved in innate immunological processes, and APMAP is involved in adipocyte differentiation [31]. Studies in SjD and non-SS salivary glands have demonstrated adipocyte infiltration into IL-6-rich areas (including IL-6-positive adipocytes) of the labial salivary gland of SjD subjects, especially those with anti-Ro60/SS-A and/or La/SS-B autoantibodies and a minor SG biopsy focus score ≥ 1. Adipocytes may be involved in salivary immune responses, and interestingly, fatty replacement of salivary glands is elevated in the salivary glands of pSjD patients. The detection of IL-17 cells, mainly in the interstitial areas of the salivary gland, around adipocytes, and within the focal infiltrates of SjD patients, implicates adipocytes in SjD’s disease progression [31,32]. 

The lack of homogeneity in the results of the identified salivary protein panels discovered in these studies presents a notable challenge in establishing a standardized panel that can be used for distinguishing pSjD from non-SS. Furthermore, the variance in the results suggests the need for research focused on the source of variation, whether methodological differences or patient diversity.

## 4. Tear Protein Profiling

Tears normally function as a lubricant for the eyes and have a protectionary function, as these secretions can neutralize irritants and microbes. One of the principal symptoms of SjD is a sensation of dryness in the eyes. This leads to moderate-to-severe discomfort for individuals with SjD and may necessitate medical intervention to manage symptoms. Additionally, the histological changes in the lacrimal glands may be reflected by the proteome of the tear fluid, which may allow for the staging and monitoring of ocular diseases. Another attractive quality of tear fluid for proteomic analysis is the fact that tears have a relatively high protein concentration of approximately 4–10 μg/μL [33], which is important, as collected tear samples are typically low in volume (less than 10 μL). 

### 4.1. Methodological Approaches Used for Tear Proteomics 

Similar to salivary proteomics, research into analyzing the proteome of tear samples has been predominantly performed with techniques that incorporate mass spectroscopy; these techniques include MALDI-TOF, SELDI-TOF, LC-MS/MS, and 2DE [34]. 

#### 4.1.1. MALDI-TOF

One study utilizing a solid-phase extraction method in conjunction with MALDI-TOF-MS was able to create a model that had an 89.3% success rate in differentiating the proteome of tears collected from controls and samples collected from individuals with various ocular diseases, thus setting a clear precedent for the potential use of MALDI-TOF in SjD samples [35]. 

#### 4.1.2. MALDI-TOF in Combination with SELDI-TOF

One study used MALDI-TOF in conjunction with SELDI-TOF to differentiate protein samples from dry-eye patients (aqueous-deficient dry eye, lipid-deficient dry eye, or a combination of these two groups) and non-dry-eye healthy controls, identifying six proteins/peptides that were altered in individuals with dry eyes. Furthermore, the authors concluded that these six proteins could serve as markers for the condition [36]. This same study was able to produce a protein panel of the six identified proteins that had a near 100% specificity for discriminating patients with dry eyes and normal controls. Patients identified with dry eyes in this manner need to be evaluated for possible SjD, which this study did not investigate. The identification of aqueous-deficient dry eye in subjects would be the basis to determine whether this panel can distinguish dry eyes in non-SjD from dry eyes in SjD [37]. As such, without further study, there is no clinical utility of this study. 

#### 4.1.3. SELDI-TOF

Studies utilizing SELDI-TOF with MS have provided evidence that this particular technique is ideal for highly accurate mass screenings of proteins and peptides in tears. One study performed using SELDI-TOF-MS found that with a “seven-peptide multimarker panel, an artificial neural network could differentiate between patients with dry eye and healthy individuals with a specificity of 90%” [38]. However, this study did not determine whether this panel could distinguish dry eyes in non-SS from dry eyes in SjD. As noted above, distinguishing individuals with dry eyes from those without dry eyes is not a clinical problem. Studies need to determine whether such evaluations of tear proteomics can differentiate SjD from non-SS dry eyes. Thus, no clinical utility was demonstrated. Another study utilizing SELDI-TOF was specifically performed comparing pSjD, non-SS, sSjD patients, and healthy controls. This work identified ten novel biomarkers as part of the protein changes reproducibly found in the SjD group. Of these ten biomarkers, seven were downregulated and three were upregulated in pSjD compared to the protein changes detected in the control groups [39]. This result gave a sensitivity of 87% and specificity of 100% when the cutoff value of the SjD down-score was set at less than 0.5 (the positive predictive value for this sample set was 100 percent). This study also found a notable inverse correlation between SjD down-scores and epithelial injury of the ocular surface in SjD patients. These observations give credence to the potential of the study of tear fluid proteomics as a noninvasive diagnostic test for SjD [39].

#### 4.1.4. LC-MS/MS

LC-MS/MS has long been used in the field of tear proteomics due to the fact that it does not require the use of analyte-directed antibodies and can study many potential markers simultaneously [40]. Li et al. utilized a nanoliquid derivative of this technique in conjunction with 2DE and MS/MS (2Dnano-LC-MS/MS) to identify 435 proteins across three sample sets (SjD with dry eyes, non-SjD with dry eyes, and healthy controls), with 182 proteins identified in SjD patients with dry-eye symptoms [41]. The researchers conducting the study analyzed tear samples that were pooled from eight subjects in each experimental group. This pooling strategy may obscure individual variations, which poses a potential challenge in extrapolating the findings to a clinical setting, especially given the heterogeneity of this patient population.

### 4.2. Novel Tear Biomarkers in SjD

In order to successfully identify unique protein biomarkers present in tear samples in patients with SjD, a comprehensive understanding of the normal proteome of tear fluid must first be reached in order to then extrapolate differences related to pathophysiological changes. To date, one of the largest proteomic studies of healthy subjects’ tear samples identified 1543 proteins, using a TripleTOF 5600 system [42]. The same study concluded with appropriate confidence that the protein IDs (exclusive identifiers given to the set of proteins that make up the proteome) generated could be used for the subsequent identification of novel biomarkers, an important foundation for the wide array of tear proteomics analyses in SjD.

#### 4.2.1. Liquid Chromatography–Tandem Mass Spectrometry Studies

One study utilizing LC-MS/MS identified the seven proteins (Ig alpha-1 chain C region, lacritin, lactoferrin, lipocalin-1, lysozyme C, polymeric Ig receptor, and a prolactin-inducible protein) that were noted in tear fluid samples collected from both non-SS dry-eyed individuals and SjD dry-eyed individuals [43]. These proteins may be involved in the dryness of the ocular surfaces associated with both SjD and non-SS dry eyes. Additionally, an increased ratio of tear MMP-9 to lactoferrin was noted and described as a distinct expression for SjD patients. This study concluded that lipocalin-1 and tear MMP-9 could serve as potential biomarkers for SjD patients. Additionally, the results generated support the use of flush tears as a staging medium for the disease process, but it should be noted that this would require lactoferrin-corrected analysis for statistical accuracy (Table 4) [43].

Another study utilizing LC-MS in conjunction with size-exclusion chromatography and sole LC-MS indicated that proteins involved with TNF-α signaling and B-cell survival were overexpressed in SjD subjects compared to healthy controls: CPNE1 and PRDX3, respectively (Table 4) [31]. This study also concluded that lipocalin was upregulated in both salivary and tear proteomics analyses. This study does not show the discriminatory potential of this technique, as non-SS was not studied. In addition, no longitudinal studies were performed, so there are no data concerning disease progression. DAVID analysis highlighted differential expression of proteins involved in protein folding in tears for SjD patients, likely correlated with the decreased tear breakup time noted in SjD corneal staining. This study also utilized nanoparticle tracking to analyze the mean particle size of tear fluid of SjD samples in order to compare these values to the control results. The findings, however, indicated no statistical differences in the mean particle size of tear fluids when comparing samples obtained from controls and pSjD patients.

#### 4.2.2. Two-Dimensional Nano-Liquid Chromatography–Tandem Mass Spectrometry 

Li et al. investigated tear fluid proteins from patients with SjD and dry-eye syndrome, subjects with dry-eye symptoms, and healthy controls using 2D-nano-LC-MS/MS (two-dimensional nano-liquid chromatography coupled with tandem mass spectrometry) [41]. This work found that patients with SjD and dry eyes had a statistically significantly increased normalized tear protein content, as well as unique proteins including defensin α1, clusterin, and lactotransferrin [41]. This study was performed using a sample size of eight for each group; thus, conclusions cannot be made until larger numbers of subjects are studied. Similar to other studies, this study found that proteins related to inflammation (both acute and chronic) were generally upregulated. Of note, Li et al. also found that proteins involved with oxidative stress injury were overexpressed—these proteins include superoxide dismutase 1 and heat shock proteins (Table 4) [41].

#### 4.2.3. Shotgun Proteomic Analysis

Das et al., utilizing bottom–up proteomics, found that alterations to protease activity are an indicator of the initiation of pro-inflammatory activation and immunological recruitment in patients with SjD [7]. This study identified 20 proteases and protease inhibitors in tear samples that were dysregulated in pSjD samples compared to healthy controls. This study also quantified and then compared proteoglycan 4 (PRG4) levels in pSjD patients compared to healthy controls and, ultimately, found that the pSjD samples had downregulated levels of PRG4. This same group had previously found that PRG4 may be related to the lubricating mechanism of tear fluid, and that pro-inflammatory proteins such as cytokines may alter its expression by epithelium cells in the cornea of the eye [7,44]. While further research is still needed, these results may provide a molecular explanation for the degeneration of the lubrication of the corneal surface of the eye associated with this disease. Additionally, similar to other studies, this study found that proteins involved with metabolism were dysregulated in the proteomes of SjD tear samples. Thus, proteomic analysis offers insights into both glandular degeneration and the dysregulation of general processes therein when studying SjD compared to healthy controls. 

#### 4.2.4. Cathepsin S Tear Film Analysis

Cathepsin S (CatS) is a cysteine endoprotease that plays a key role in the degradation of the MHC II invariant chain (li). Regarding the enzyme’s significance to autoimmunity, early studies, such as those performed by Saegusa et al., found that CatS inhibitors could inhibit the autoantigen-initiated T-cell proliferation response. CatS dysregulation has been linked to the overactivation and exaggeration of many inflammatory chemokines, and inhibition of these enzymes in SS mouse models has shown that CatS may be involved with the autoimmunity associated with SS; thus, CatS may be a target of proteomic analysis as a salivary and tear film biomarker [45]. 

Studies conducted on murine models of SjD have also shown that tear film proteome analysis can be used to accurately assess CatS activity, allowing for the creation of synthetic CatS inhibitors. One study using proteomic analysis of tear films collected from mice found that CatS inhibition resulted in decreased lymphocytic infiltration into the lacrimal glands, with increased stimulated tear secretion [46]. Ultimately, these murine and ex vivo studies, along with the demonstration of the safety of these inhibitors in animal models, contributed to recent studies using human subjects.

The theoretical association between CatS activity and the ocular characteristics that were demonstrated in murine models was examined by Klinngam et al., in a study that highlighted an association between increased simulated CatS activity and several key markers of surface inflammation, such as IL-6, TNF-α, IL-8, and Il-1β [47].

One study performed on human subjects, utilizing ELISA analysis of tear samples from 15 pSjD patients and 13 controls, found that CatS was greatly upregulated and inhibition of its activity led to a decrease in the autoantigenicity of T cells and pSjD [48]. This research indicates the need for further research conducted with larger sample sets examining the relationship between CatS inhibition and disease pathogenicity, as well as the validity of CatS as a novel biomarker.

Another study aiming to identify the potential use of CatS tear film activity as a diagnostic tool found that CatS activity was significantly elevated in the tears of SjD patients when compared with rheumatoid arthritis, SLE, and other autoimmune conditions sans sSjD. These findings highlight the need for further studies comparing CatS tear activity in SjD, sSjD, and non-SS patient populations [49]. One other such study also found that CatS activity was markedly increased in pSjD patients, and in ex vivo models the CatS inhibitor RO5459072 can be used as a dose-dependent suppressor of the exaggerated T-cell response found in SS [50].

These results highlight the potential of CatS inhibitors as a novel therapeutic particularly targeting the dry-eye symptoms of SjD. However, one study recently investigated the clinical use of CatS inhibitors for pSjD and found that there were no significant improvements in the experimental group when administered [51]. The study was consistent with earlier findings that the experimental therapy elicits a decrease in the circulation of both B cells and T cells; however, the researchers ultimately found this difference to be statistically nonsignificant. This demonstrates the need for future parallel studies to examine the validity of CatS upregulation as a biomarker. However, given the plethora of studies that have implicated the activity of CatS in the ocular symptoms associated with SS, this enzyme warrants more tear film proteomics research. Additionally, this study suggests that CatS upregulation as a biomarker in and of itself may not be sensitive enough to distinguish SjD patients from dry-eyed non-SS patients; however, another study found that CatS activity in conjunction with lactoferrin may allow for this distinction [52].

## 5. Conclusions

This review illustrates the potential use of proteomic methods for diagnosis and monitoring of the condition in a less invasive manner than traditional serological methods. Many studies have compared findings from SjD patients and healthy controls, or between dry-eye patients and healthy controls. However, there is no practical clinical utility in studying salivary/tear proteomes between SjD patients and healthy controls. Nonetheless, some research in salivary and tear proteomics also indicates that proteomic analysis may allow for the accurate distinction of SjD patients from non-SS patients; this represents a significant advantage of this approach, as this would allow for better classification of patients for clinical treatment and research. Overall, however, we conclude that proteomic analysis is proceeding towards a more holistic understanding of the molecular etiology of the disease—specifically, the significance of the activation of various biochemical pathways involved in inflammation, lymphocyte recruitment and survival, and the degeneration of proteins involved in overall saliva and tear composition and secretion. The plethora of independent studies in this field certainly highlights the efficacy of the proteomic approach for identifying alterations in proteome composition as a reflection of glandular degeneration; however, research regarding the accuracy of the use of these protein biomarkers as a diagnostic medium is not (at this point in time) comprehensive enough for real-world clinical applications. For example, no study has included exploratory and confirmatory groups. Furthermore, while studies using similar or highly related techniques have found differences, these differences have not been reproduced between studies. Currently, several questions remain regarding the use of protein biomarkers for SS identification, including “What protein biomarker or set of proteins would be the best candidate for diagnosis using proteomic analysis”, “Are individual protein biomarkers or protein panels comprised of several differentially expressed proteins more accurate for identifying SS”, “What methodological approach is best suited for standardization of the proteomic approach”, “How does the accuracy of diagnosis using proteomic panels or biomarkers compare to traditional serological methods”, and “What is the feasibility of standardizing a formal staging schema for the disease using proteome alterations?”.

## Figures and Tables

**Table 1:** AECG classification criteria for SjD (2002).

Items	I. Ocular symptoms (at least one present)	Persistent dry eyes for more than 3 months Sensation of sand or gravel in eyes Use of tear substitute more than 3 times a day
	II. Oral symptoms (at least one present)	Persistent dry mouth for more than 3 months Recurrent swollen salivary glands as an adult Use of fluids to aid with swallowing dry foods
	III. Objective evidence of dry eyes	Schirmer’s test ≤ 5 mm/5 min or van Bijsterveld score ≥ 4
	IV. Objective evidence of salivary gland involvement (at least one present)	Unstimulated whole salivary flow ≤ 0.1 mL/min Parotid sialography Salivary gland scintigraphy
	V. Histopathological evidence	Focus score ≥ foci/4 mm^2^ from minor salivary gland biopsy
	VI. Serological abnormality	+Anti-SSA or SSB antibodies
Rules for classification	1. Absence of AECG exclusion criteria ^a^ 2. Four items must be present for classification; one of these items must be either V or VI	

^a^ Exclusion criteria for AECG classification: acquired immunodeficiency syndrome, history of head and neck radiotherapy, hepatitis C, graft-versus-host disease, pre-existing lymphoma, sarcoidosis, and the use of anticholinergic drugs within a time period shorter than fourfold the half-life of the drug.

**Table 2:** ACR/EULAR classification criteria for SS (2016).

		Weight/Score
Items	Labial salivary gland with focal lymphocytic sialadenitis and focus score of ≥1 foci/ 4 mm ^2^	3
	2. Anti-SSA/Ro-positive	3
	3. Ocular staining score ≥ 5 (or van Bijsterveld score ≥ 4) in at least one eye	1
	4. Schirmer’s test ≤ 5 mm/5 min in at least one eye	1
	5. Unstimulated whole saliva flow rate ≤ 0.1 mL/min	1
Rules for classification	1. Absence of exclusion criteria ^a^ 2. Has at least one symptom of oral or ocular dryness, or ESSDAI ≥ 1 3. Weight/score of ≥4	

^a^ Exclusion criteria for the ACR/EULAR criteria: acquired immunodeficiency syndrome, history of head and neck radiotherapy, hepatitis C, active tuberculosis, graft-versus-host disease, sarcoidosis, amyloidosis, and IgG4-related disease processes.

**Table 3:** Summary of proteomic analysis of pSjD patient saliva samples.

Experimental Method	Differentially Expressed Proteins Identified
Mass spectrometry in tandem with Western blotting and enzyme-linked immunosorbent assay (ELISA)	**↑** α-Enolase, IGKC, psoriasin (protein S100-A7), calgranulin B (protein S100-A9), E-FABP, beta-2-microglobulin, **↓** α-Amylase precursor, carbonic anhydrase VI, G3PDH, PIP, SPLUNC-2
Liquid chromatography–tandem mass spectrometry results were analyzed with DAVID and the Functional Enrichment Analysis Tool (FunRich)	**↑** Secreted Ly-6/uPAR-related protein 1, beta-2-microglobulin, clusterin
Liquid chromatography and tandem mass spectrometry ^a^	**↑** Neutrophil elastase, calreticulin, tripartite motif-containing protein 29
Liquid chromatography and tandem mass spectrometry	**↑** DJ-1/Parkinson disease protein 7, cathepsin G, neutrophil elastase, lactotransferrin, azurocidin, cystatin-SA, calpastatin, proteinase 3/myeloblastin, alpha-1-antitrypsin, transmembrane protease serine 11D

^a^ This study identified 40 differentially expressed proteins in the saliva of pSjD samples; however, this table depicts the three proteins that were found to be the best candidates for novel biomarker detection of pSjD; **↑** denotes overexpression; **↓** denotes under expression.

**Table 4:** Summary of proteomic analysis of pSjD patient tear samples.

Experimental Method	Differentially Expressed Proteins Identified
Liquid chromatography–tandem mass spectrometry	**↑** MMP-9:lactoferrin **↓** Lipocalin-1, lacritin, prolactin-inducible protein
Liquid chromatography–tandem mass spectrometry in conjunction with size-exclusion chromatography and sole LC-MS	**↑** Neutrophil gelatinase-associated lipocalin, CPNE1, PRDX3
Two-dimensional nanoliquid chromatography–tandem mass spectrometry	**↑** CDNA FLJ78387, annexin A2 isoform 1, serotransferrin precursor, keratin 4, protein S100-A9, mucin-5AC precursor, annexin A1, keratin type I cytoskeletal 10, keratin type II cytoskeletal 1, protein S100-A8, complement C3 precursor, actin, **↓** Growth-inhibiting protein 12, lipocalin-1 precursor, prolactin-inducible protein precursor, IGHA1 protein, polymeric immunoglobulin receptor precursor, extracellular glycoprotein lacritin precursor, lysozyme C precursor, proline-rich protein 4 precursor, cystatin-S precursor, keratin type II cytoskeletal 5
High-performance liquid chromatography and mass spectroscopy	**↑** Matrix metalloproteinase-9, cystatin-D, neutrophil collagenase, cystatin-C, cathepsin B, leukotriene A-4 hydrolase, prostasin **↓** Calpain-1, cytosolic non-specific dipeptidase, kininogen-1, alpha-2-antiplasmin, DJ-1/Parkinson disease protein 7, proteasome subunit alpha type-6, proteasome subunit alpha type-3, acylamino-acid-releasing enzyme, proteasome subunit type-2, cytosol aminopeptidase, phosphatidylethanolamine-binding protein, proteasome subunit beta type-8, leukocyte elastase inhibitor

**Note: **↑**** denotes overexpression; **↓** denotes underexpression.
